# Publisher Correction: Shape and fluctuations of frustrated self-assembled nano ribbons

**DOI:** 10.1038/s41467-019-12300-8

**Published:** 2019-09-13

**Authors:** Mingming Zhang, Doron Grossman, Dganit Danino, Eran Sharon

**Affiliations:** 1 The Racah institute of Physics, The Hebrew University of Jerusalem, Jerusalem, Israel; 2 CryoEM Laboratory of Soft Matter, Faculty of Biotechnology and Food Engineering, Technion—Israel Institute of Technology, Haifa, Israel

**Keywords:** Self-assembly, Nonlinear phenomena

Correction to: *Nature Communications* 10.1038/s41467-019-11473-6, published online 8 August 2019.

In the original version of this Article, reference 34 was incorrectly given as: “Danino, D. Emerging microscopy methods in colloid science. *Curr. Opin. Colloid Interface Sci*. **17**, 316–329 (2012)” instead of the correct “Danino, D. Cryo-TEM of soft molecular assemblies. *Curr. Opin. Colloid Interface Sci*. **17**, 316–329 (2012)”. This has been corrected in both the PDF and HTML versions of the Article.

