# Supplementary material for: Shape and fluctuations of frustrated self-assembled nano ribbons
Source: Nat Commun. 2019 Aug 8;10:3565. doi: 10.1038/s41467-019-11473-6 (PMC6687827; doi:10.1038/s41467-019-11473-6)
Supplement: Supplementary file 1 — Supplementary Information [file 41467_2019_11473_MOESM1_ESM.pdf]

Supplementary Information for "**Shape and Fluctuations of Frustrated Self Assembled Nano Ribbons**"

By Zhang et.al.

Supplementary Methods

The molecular structure of the C_{12}-β_{12} is shown in Fig. 1. The molecule, a member in the OAK family (*1, 2*) was synthesized and purified as previously described (*3*) and stored in dry form. C_{12}-β_{12} solutions at desired concentrations were prepared by dissolving the powder in Milli-Q water, vortexing and brief sonication, followed by two hours incubation at 90 °C, then incubation at room temperature (25 °C).

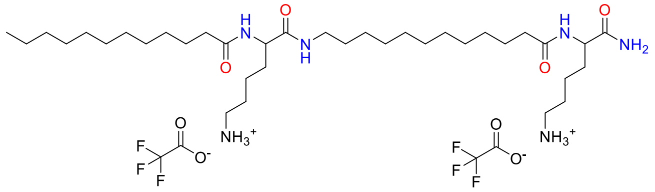

Supplementary figure 1: The C_{12}-β_{12} (N-α-lauryl-lysyl-aminolauryl-lysyl-amide) molecular structure.

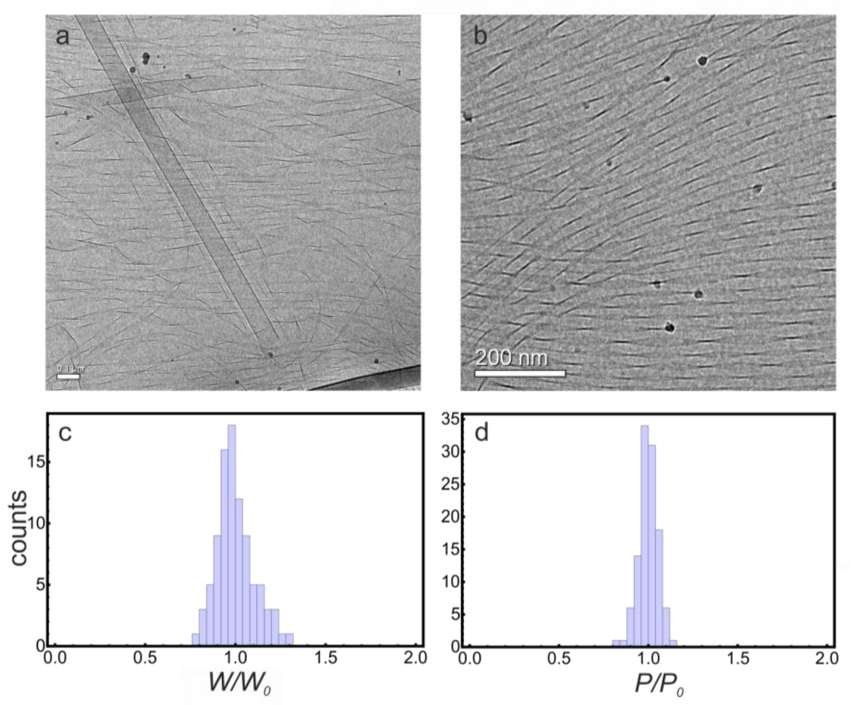

Supplementary figure 2: Cryo-TEM images and dimensionless analysis of ribbons' width and pitch. **(a)** A low magnification image of a C_{12}-β_{12} sample aged for ~2 months. Typically, at this stage ribbons of different geometry and width populate the sample. **(b)** A low magnification image of a C_{12}-β_{12} sample after 2 days of incubation. This sample is populated with long twisted ribbons of different widths. The width and pitch along a given ribbon are in general nearly constant. **(c)** Normalized width, and **(d)** normalized pitch distributions within individual twisted ribbons. The local width and pitch measurements were normalized by the values averaged over each ribbon.

Supplementary notes

1) Emergence of twist from molecular chirality: Close packing of the head groups can provide estimation to the preferred conformation of neighboring head groups. Here, hydrogen bonds between adjacent head groups (bond energy about 22KJ/mol (*4, 5*) pull adjacent molecules closer than the preferred lysine head group size ($\gtrsim 0.6$ nm) (*3*).

A simplified model for an amphiphile (not C_{12}-β_{12}) monolayer is shown in Figs. S3-S5. The molecules are composed of a long hydrocarbon tail and an amide group, connected to a chiral carbon, along with three other general functional groups shown as white, green and orange spheres. Spheres of different sizes represent the groups' Van der Waals (VdW) volume. Each two molecules are connected via a hydrogen bond (cyan line) between the amide groups. In this model (as in the C_{12}-β_{12} assemblies), the length of the hydrogen bond is smaller than the total size of the head groups. Therefore, head groups will pack as closely as possible, given their geometry (group's size and position). In order to do so it is clear that the head must develop a twist angle, which depends on both the head geometry and tail length (see calculation in supp. equation 15). Here we focus on understanding the preferred twist angle of the heads alone θ_0, without considering the tails.

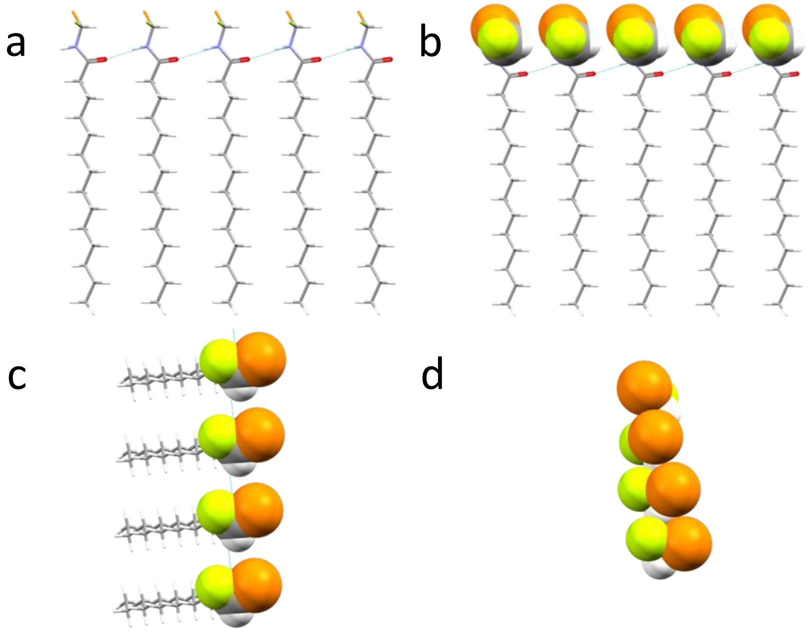

Supplementary figure 3: Induced twist via close packing. Figure is showing the configuration of simple chiral amphiphiles in the self-assembly of left-handed ribbons. A single molecule is composed of a long hydrocarbon tail and an amide group. When assembled, hydrogen bonds form between adjacent amide groups (dotted cyan lines). Assemblies are visualized (side view) using capped sticks model **(a)**, space fill style head groups **(b)** and top view **(c)**, and twisted close packing of headgroups only (tails and chiral carbon are transparent) **(d)**. Green, orange and white balls represent the three groups which are connected to the chiral carbon. Ball volume represents relative VdW volume of each group.

As an initial step, consider a non-chiral head group consisting of three same-type groups around a central atom. A close packing of such a system may be achieved by twisting each head group by 60° (either

clockwise or counterclockwise). Now, consider a modified head group such that all 3 sub-groups are different. In this case, close packing is achieved by a twist angle which is not $60°$, but will typically remain within $10° < \theta_0 < 60°$ (unless there is a huge size difference).

The handedness of the packing depends on the chirality of the carbon, as it breaks the mirror symmetry (see Fig. S4). Thus a specific handedness is chosen such that the largest head group (orange sphere), being the most significant limiting factor, fits into the largest space between head groups in the neighboring molecule. This condition ensures that we may pack the heads as close as possible.

As an example, consider the hypothetical head group in Fig. 4, composed of four different groups (orange, green-yellow, blue and white) around a central group (grey). When the next one is assembled (towards the viewer) the hydrogen bond direction is along the line connecting the white group of the lower one and the grey group of the upper one, causing them to close-pack. As a result, the molecules form a twist between them which may be a priori either clockwise or anti-clockwise. A *clockwise* rotation (corresponding to a right-handed twist) is illustrated in Fig. 4 (b) and (d). Such conformation results in a large overlap of the projection (the shadowed region) between the largest (orange) group of one molecule and the other (the semitransparent orange) molecule, implying a large distance between the molecules. *Anti-clockwise* rotation (corresponding to a left-handed twist) is illustrated in Fig. 4 (a) and (c). The overlap is smaller, making it possible for the orange group to fit better in the gap (between orange and blue groups of the first molecule). Therefore a left-handed twist is preferred in this molecule (Fig. S5 B middle). For the enantiomer of this molecule, a right-handed twist is preferential (Fig. 5 A middle).

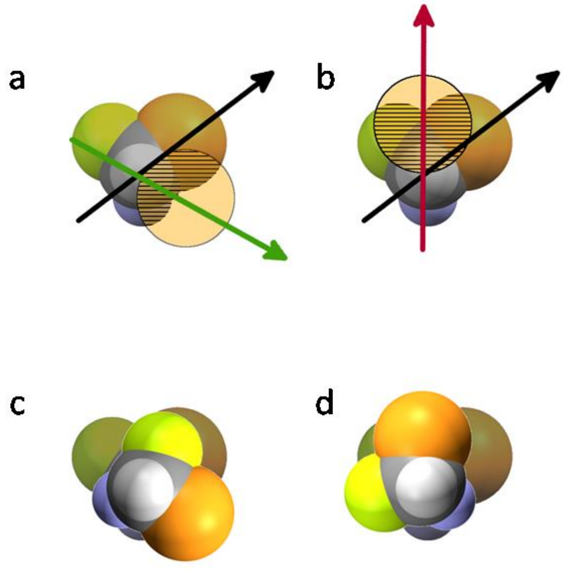

Supplementary figure 4: When a second molecule is attached from above (the bond is at the middle of white group), it will orient such that the largest subgroup (orange) fits into the largest space available. As can be seen, in subfigure **(a)** there is less occupied volume (shaded area) at the position of the orange group, allowing to molecules to align closer. Therefore left-handed configuration (**a** and **c**) is preferential over right-handed configuration (**b** and **d**). Arrows mark the orientation of the molecules.

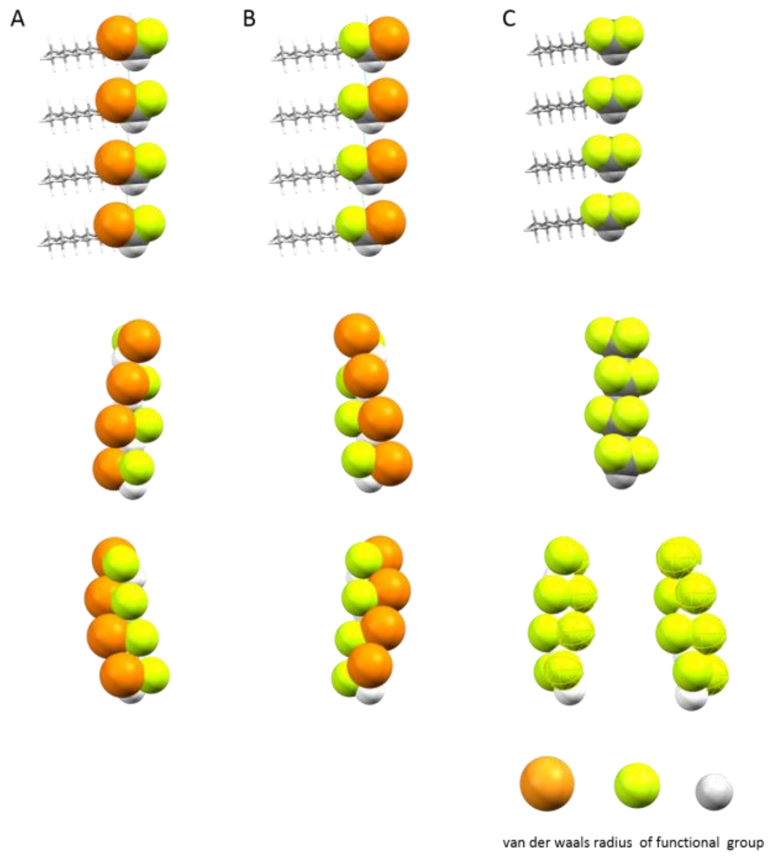

Supplementary figure 5: Preferred handedness induced by chiral carbon. An illustration of an assembled column of amphiphilic molecules. The molecules in **(A)** and **(B)** have opposite chiral carbon in their head groups, determined by the cyclic order of distinguished side groups (in this case, side groups have different VdW radius). The molecule in **(C)** has an achiral head group, since it has two identical side groups. An illustration of a right handed packing of the **A** head groups (**A** - middle panel), which is mirror-symmetric to the left handed packing of **B** (**B** - middle panel), but not to the left hand packing of **A** (**A** bottom panel). Following the rational given above, the configurations in the middle panels (both **A** and **B**) will be energetically preferable over those in the bottom panels. The combination of chiral heads and close packing, therefore, leads to twist with a selected handedness. The handedness flips a sign upon changing the chirality of the head group. In contrast, the left and right handed packing of molecule in **(C)** are mirror symmetric. Therefore, they must have the same energy. In fact, even a zigzag packing is energetically equivalent, due to local (between neighboring molecules) mirror symmetry.

The C_{12}-β_{12} molecule is much more complicated than the illustrations in Fig. 3-5. Still, the principles of close packing, together with strong chirality (large difference in VDW volumes) of the head groups induce a preferred twist between head groups: Considering a molecule which contains (S) chiral carbon (Fig. S6) and asking where is the largest available volume for the methylene group (as the nearest part of lysine head group, $D \sim 0.6\ nm$), we note (Fig. S6) that the neighboring oxygen atom (C=O group, black arrow) is significantly larger than the opposite hydrogen (N-H group, green arrow). Therefore, a right-handed twist (marked by green arrow (Fig. S6 b)), which yields a tighter packing, is preferred. Indeed, the chirality of the C_{12}-β_{12} self-assembled nanoribbons is right-handed as indicated by SEM (Fig. 7).

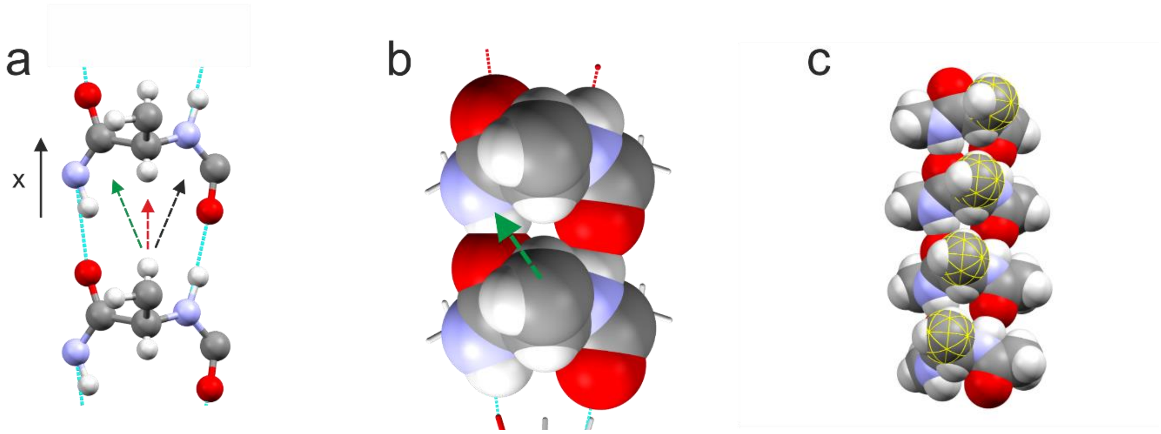

Supplementary figure 6: Preferred twist by head group chirality in C_{12}-β_{12} sheets. **(a)** The configuration of two (chiral) heads of the molecules. The cyan dotted lines mark the hydrogen bonds formed by adjacent amide groups. The red, blue, gray and white sphere represent atom of oxygen, nitrogen, carbon and hydrogen respectively. Close packing will cause tilt of the lower molecule, either to the left (green arrow) or to the right (black arrow). **(b)** The head groups in (a) illustrated with volumes proportional to the Vander Waals volume of each group. The volume of hydrogen (left) is smaller than oxygen (right), therefore the molecules will be twist to the left (green arrow). **(c)** An illustration (not a computation) of the preferred packing. The strong right handed twist along the x direction is clear.

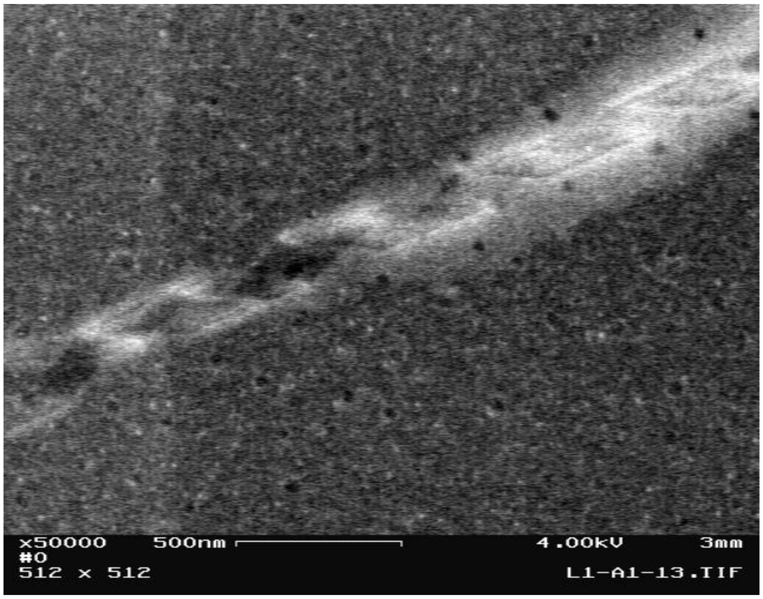

Supplementary figure 7: HR-SEM images of right-handed coiled ribbons constructed by C_{12}-β_{12}, Scale bar = *500nm*. In SEM technique, a fine electron beam (probe) scans the specimen and an image is formed from the resulting scattered electrons that are emitted and reflected from the specimen surface. Therefore, (unlike TEM images) the images clearly distinguish between the up/down surfaces of the helix and can be used to detect the handedness of ribbons.

2) Estimations of the reference twist and reference curvature: The reference twist, which is a component of the reference curvature tensor is set by the local interactions between molecules. While such interactions can be simulated in order to obtain accurate values, here we estimate them in order to obtain approximated values and scaling laws. As in (*6*) we express the "two molecular energy" as a sum of two terms: The head groups interaction is quadratic in the angular deviation from the optimal (close packing) angle, θ_0: $E_{head} = \frac{1}{2}\gamma D^2(\theta - \theta_0)^2$, where γ is a "spring constant" and D is the head group diameter. We use estimations of the preferred head groups twist angle $10° < \theta_0 < 60°$, the interaction energy of $80\frac{KJ}{mol}$ (*4, 5*), and the head group size $D \cong 0.6$ nm (*3*). Similarly, the carbon chains prefer a perfectly aligned orientation, thus the energetic modeling is $E_{CC} = \frac{1}{2}\beta L^3\theta^2$, where $L \cong 3.4$ nm is the carbon chain length and βL is the effective spring constant between both carbon chains. Notice the different scaling of the energy terms: In the head groups interaction, the "spring constant" is independent of the head size (and depends on the type of hydrogen bond). For the carbon chains the (Van der Waals) attraction between carbon atoms increases with the number of molecules in the chain. Thus the "spring constant" itself depends on L. In C_{12}-β_{12}, the chains consists of 22 methylene groups $(-CH_2-)$ with a total energy of $\approx 40-60\frac{KJ}{mol}$ (*4, 5*). The additional dependence of the energy terms on D and L comes from the fact that the typical deviation (length) from preferred configuration is merely the angle difference times the size of the head/carbon chain (the larger the heads/ carbon chain, the more distant they get for the same deviation angle). For a single line of amphiphiles, we therefore get the "reference twist angle", by balancing these two energies, i.e- solving the equation

$$0 = \frac{\partial E_{Tot}}{\partial\theta} = \frac{\partial E_{head}}{\partial\theta} + \frac{\partial E_{CC}}{\partial\theta} = \gamma D^2(\theta_{eq} - \theta_0) + \beta L^3\theta_{eq} \quad (\text{eq. }1)$$

Whose solution is

$$\theta_{eq} = \frac{\gamma D^2\theta_0}{\gamma D^2 + \beta L^3} \quad (\text{eq. }2)$$

Note that this expression provides the dependence of the "force constants", presented in (*6*) (Eq. 18), on the relevant geometrical dimensions of the molecules, i.e. *L* and *D*. Using Mercury CSD (The Cambridge Crystallographic Data Centre) (*4, 5*), we find that

$\gamma \simeq \frac{16}{0.3}\frac{KJ}{mol \cdot nm} \sim \beta L$. Also $D = 0.6$ nm and $L \cong 3.4$ nm. Hence $\frac{\gamma D^2}{\gamma D^2+\beta L^3} \cong \frac{1}{33}$ resulting with

$$0.3° < \theta_{eq} < 2°\,.$$

In turn, this implies that $k_0 \cong \frac{\theta_{eq}}{D}\frac{rad}{nm}$ is $0.008 < k_0 < 0.058$. By estimating the typical length difference of a primary and secondary amine bonds (*7*) (Δd), the group head-size (D), and carbon chain length (L), we may calculate

$$\alpha k_0 \sim \frac{\Delta d/D}{L} = \frac{0.003/0.6}{3.4} = \frac{0.005}{3.4} = 0.0014 \xrightarrow{\text{yields}} 0.02 < \alpha < 0.17$$

We thus use the estimation for $\alpha = 0.11,\ k_0 = 0.03\ (nm^{-1})$, when comparing the data to the theory in the main text.

3) Theoretical Background - Incompatible elastic ribbons: Incompatible ribbons are thin elastic sheets whose internal geometry does not comply with that of three dimensional Euclidean space in which it is embedded. The elastic theory of incompatible sheets was derived in (*8*). Within this framework, the elastic sheet's energy depends on deviations of its metric a, which describes distances between neighboring material elements, from the sheet's reference metric $\bar{a}$, (which describes preferred distances). Similarly, deviations of the curvature b from the reference curvature $\bar{b}$ also contribute to elastic sheet's energy. The energy of a narrow ribbon, in which the width is much smaller than the length, was developed in (*9*) by means of dimensional reduction. It estimates the two dimensional energy functional by approximating the shape of the ribbon using the curvatures at its mid-line only. It is a general Hamiltonian describing both compatible ribbons and incompatible ribbons of many types and different internal geometries. In the case of ribbons with spontaneous twist and a Euclidean reference metric it is given by

$$E = \int H ds = \int \frac{Y}{8(1-\nu^2)}\left(\frac{1}{80} t W^5 (ln - m^2)^2 \right.$$
$$\left. + \frac{1}{3} t^3 W\big((l + n - \alpha k_0)^2 - 2(1-\nu)(l(n - \alpha k_0) - (m - k_0)^2)\big)\right) ds,$$

where l is the normal curvature along the midline, m is the twist around the midline, and n is the normal curvature perpendicular to the midline (Fig S8), Y is Young's modulus, ν Poisson's ratio, t the ribbon's thickness, and W its width (s is the position along the midline and the integral is taken over the whole ribbon).

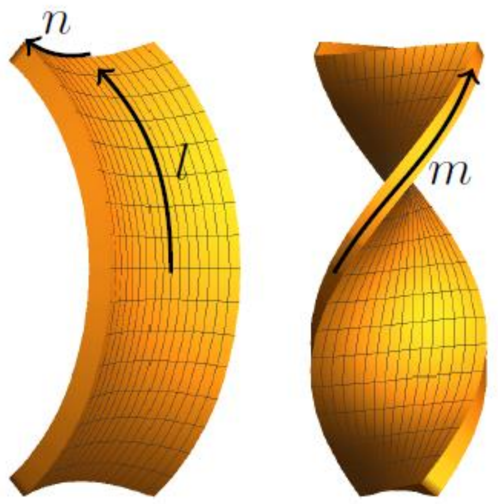

Supplementary figure 8: Visualization of the geometrical meaning of l, m, n (the curvature tensor elements). l is the normal curvature along the ribbon's mid-line; n is the normal curvature in the perpendicular direction; and m is the twist around the mid-line.

The equations describing the equilibrium values are derived by taking the partial derivatives of H by the different curvatures (l, m, n), and equating them to 0. By solving them we find that

$$l(W;\alpha,k_0,t) = \frac{1}{3(1+\nu)(1-\nu)^2\alpha\, {k_0}^3 W^4}\Big[640(1-\nu)^2{k_0}^2t^2$$
$$-(1-\nu)\left(640(3-\nu)t^2+3(1+\nu)\alpha^2{k_0}^2W^4\right)k_0m$$
$$+\left(1280(1-\nu)t^2-3(1+\nu)^2\alpha^2{k_0}^2W^4\right)m^2-6(1-\nu)(4+\alpha^2)k_0W^4m^3$$
$$+12(4+\alpha^2)W^4m^4\Big] \quad (eq.S1)$$

$$n(W;\alpha,k_0,t) = \frac{1}{3(1+\nu)(1-\nu)^2\alpha\, {k_0}^3 W^4}\Big[640(1-\nu)^2{k_0}^2t^2$$
$$-(1-\nu)\left(640(3-\nu)t^2+3\nu\,(1+\nu)\alpha^2{k_0}^2W^4\right)k_0m$$
$$+\left(1280(1-\nu)t^2-3(1+\nu)^2\alpha^2{k_0}^2W^4\right)m^2-6(1-\nu)(4+\alpha^2)k_0W^4m^3$$
$$+12(4+\alpha^2)W^4m^4\Big] \quad (eq.S2)$$

$$m(W;\alpha,k_0,t) = Root\Big(-80(1-\nu)^3{k_0}^3t^2+80(1-\nu)^2(5-\nu){k_0}^2t^2x-4(1-\nu)(2-\nu)80k_0t^2x^2$$
$$+\left(320(1-\nu)t^2+3(1-2\nu-\alpha^2\nu+\nu^2){k_0}^2W^4\right)x^3-3(1-\nu)(4+\alpha^2)k_0W^4x^4$$
$$+3(4+\alpha^2)W^4x^5\Big) \quad (eq.3)$$

Note, the equations for l and n differ very slightly (by the second term in the second row), also note that while both seem to depend on m, for a given (t, W, k_0, α), m is merely a "number" (i.e. the root of equation (3)).

We are interested in the pitch P, and radius R, which are formally given by:

$$R(W;\alpha,k_0,t) = \frac{l(W;\alpha,k_0,t)}{l^2(W;\alpha,k_0,t)+m^2(W;\alpha,k_0,t)}, (eq.4)$$

$$P(W;\alpha,k_0,t) = \frac{2\pi\, m(W;\alpha,k_0,t)}{l^2(W;\alpha,k_0,t)+m^2(W;\alpha,k_0,t)}, \quad (eq.5)$$

For $\alpha \neq 0$, we can expand these *solutions in the narrow limit ($W^2 \ll \frac{t}{k_0}$), to find:*

$$R_{narrow} = \Xi W^4$$

$$P_{narrow} = \frac{2\pi}{k_0} + \frac{3\pi k_0}{40(1-\nu)t^2}W^4$$

where $(t, \nu, \alpha, k_0) = \frac{3(16+\alpha^2(1+\nu)^2\, k_0}{80\, t^2\alpha\,(1-\nu)^2(1-\nu^2)}$. The wide limit is given in the main text.

For $\alpha = 0$ equations (4, 5) simplify, as the solutions coincide with those computed in (*10*). And are given by

$$P(W,k_0,t) = \frac{3\, 5^{\frac{1}{3}}\pi W^2\left((1-\nu)\left(9W^2k_0+\sqrt{81\,k_0^2W^4+320(1-\nu)t^2}\right)\right)^{\frac{1}{3}}}{\left(5^{\frac{2}{3}}\left((1-\nu)\left(9W^2k_0+\sqrt{81\,k_0^2W^4+320(1-\nu)t^2}\right)\,t^2\right)^{\frac{2}{3}}-20(1-\nu)\,t^{\frac{4}{3}}\right)}, (eq.6)$$

$$R(W, k_0 t) = 0\,, \qquad (eq. 7)$$

For $W < \left(\frac{320}{3}\frac{(1+\nu)}{(1-\nu)^2}\frac{t^2}{k_0^2}\right)^{\frac{1}{4}} = W^*$ *so that in the narrow limit* $(R_{narrow}(\alpha = 0) = 0)$

$$P(W, k_0 t) = 2\pi \frac{3(1-\nu)k_0\, W^4}{3\,(1-\nu)^2 k_0^2\, W^4 - 160(1+\nu)t^2}, \qquad (eq. 8)$$

$$R(W, k_0 t) = \frac{W^2\sqrt{9\,(1-\nu)^2 k_0^2\, W^4 - 960(1+\nu)t^2}}{3\,(1-\nu)^2 k_0^2\, W^4 - 160(1+\nu)t^2}, \qquad (eq. 9)$$

for $W \geq \left(\frac{320}{3}\frac{(1+\nu)}{(1-\nu)^2}\frac{t^2}{k_0^2}\right)^{\frac{1}{4}} = W^*$.

This case ($\alpha = 0$), was discussed in details in (*9*), the solution describes a sharp continuous transition between twisted and helical structures at a critical width W^*. Above the critical width, there are two stable solutions to the problem, differing in the choice of orientation of the ribbon (though physically describing the same configuration up to a rigid body rotation). This happens due to symmetry between the (absolute) values of the principal curvature. The general case of $\alpha \neq 0$, corresponds to a broken symmetry, hence we would expect smooth and more gradual transition as α gets further away from 0, while the system prefers one solution over the other. As seen in Fig. 9, this is indeed the case. One can also see from Fig. 9 that the "transition" (which now may be defined as the position of the peak in the pitch graph, or the largest derivative in the radius graph) occurs earlier for the same values of k_0, ν. This is due to the fact that larger α values correspond to larger mean reference curvature. For this reason, also the final maximal radius decreases with α, hence producing a larger pitch angle (Fig. 3b main text).

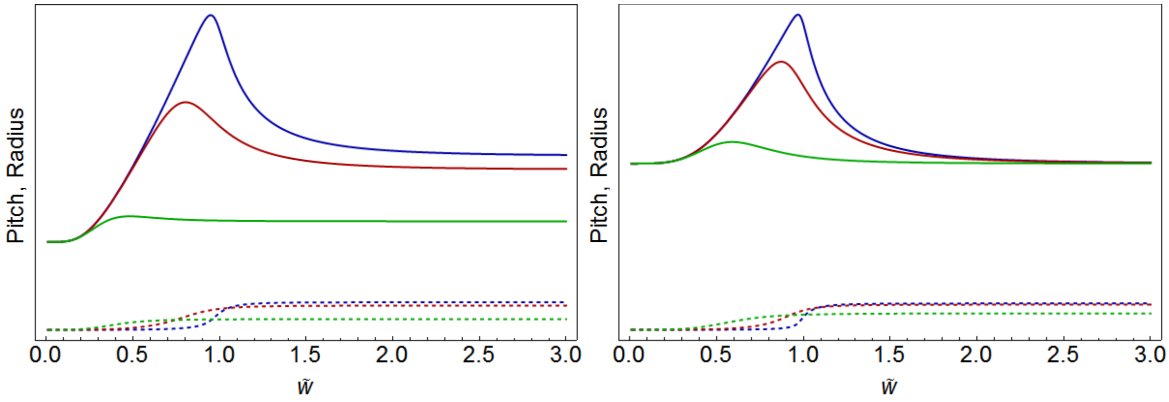

Supplementary figure 9: Dimensionless Pitch (solid lines) and Radius (dashed) plots for different values of α (0.01- Blue, 0.1- Red, 1- Green) for different values of the Poisson's ratio, ν (0.5 left, 0 right).

4) Thermal fluctuations: For a ribbon in a thermal environment, the probability of finding a shape which is not its equilibrium shape is such that

$$p \propto e^{-\frac{\Delta E}{k_B T}} \quad (eq. 10)$$

Where ΔE is the (elastic) difference between the energy of the configuration and the energy of the equilibrium shape, T is the surroundings' temperature, and k_B is Boltzmann's factor. As described in (*9*), for small enough fluctuations we may expand the Hamiltonian to second order in the fluctuations, which simplifies the integrals significantly. We therefore change variables to a local pitch and radius variables and continue by writing:

$$\Delta E \simeq \int (\Delta\sigma(s))^T H_{\sigma\sigma}(\Delta\sigma(s))ds \quad (eq. 11)$$

Where $(\Delta\sigma) = \binom{\Delta P}{\Delta R}$, $H_{\sigma\sigma}$ is the Hessian (partial derivative matrix) of H. Since the Hamiltonian as given above, does not contain any derivatives. There are only local correlations, that is, the resulting correlations are a delta function (i.e- of the form: $\langle \Delta P(s)\Delta P(s')\rangle = H_{PP}^{-1} \propto \delta(s-s')$). When comparing to measured values, it is important to note, that measurements are not perfectly local. Rather, they are averaged over a portion of the ribbons whose length equals the ribbon's pitch. That is,

$$\Delta E = \int_{L_{link}} (\Delta\sigma(s))^T H_{\sigma\sigma}(\Delta\sigma(s))ds = L_{link}\, (\Delta\sigma)^T H_{\sigma\sigma}(\Delta\sigma) \quad (eq. 12)$$

where for a narrow ribbon $\mathrm{L_{link}} = \mathrm{P}$.

Following this exact procedure, we may integrate out radius fluctuations (ΔR), and write (e.g. for the narrow regime)

$$\Delta E = Y\, f(W) \Delta P^2 \quad (eq. 13)$$

Where Y is Young's Modulus (the elastic constant), ΔP is the average fluctuation over a segment of size P, and $f(W)$ is some non trivial function of the width, W, that has units of length. It depends only on the geometry of the problem (t, ν, α, k_0) and the width. In Fig S10, this function is plotted for $\alpha = 0.01, 0.1, 1$ as a function of the width.

$$\begin{aligned}\frac{f(W)}{tWP} = {}& ((l^2+m^2)^4(-81(m^2-ln)^3W^{12} + 512000t^6(-1+\nu)^2(1+\nu) + 720(m^2-ln)t^2W^8(l^2 \\ & + n^2 + ln(3-7\nu) + m^2(-1+7\nu)) - 19200t^4W^4(-1+\nu)(l^2+n^2+m^2(3+5\nu) \\ & - l(n+5n\nu))))/(1920\pi^2(-1+\nu^2)(9(-m^2+ln)(-m^2(l^4-10l^2m^2+m^4) \\ & + 3l(l^2-m^2)^2n)W^8 - 240t^2W^4(l^6+(l^2-m^2)^2n^2+l^2m^4(25-12\nu)+2m^6\nu \\ & + 2l^4m^2(3+\nu) - 4ln(-2l^2m^2(-2+\nu)+m^4(-2+\nu)+l^4\nu)) \\ & + 6400t^4(-2l^2m^2(-3+\nu)(-1+\nu)+l^4(-1+\nu^2)+m^4(-1+\nu^2)))) \qquad (eq. 14)\end{aligned}$$

where $(\mathrm{l}, \mathrm{m}, \mathrm{n})$ were given above as the equilibrium solutions (equations 1-3).

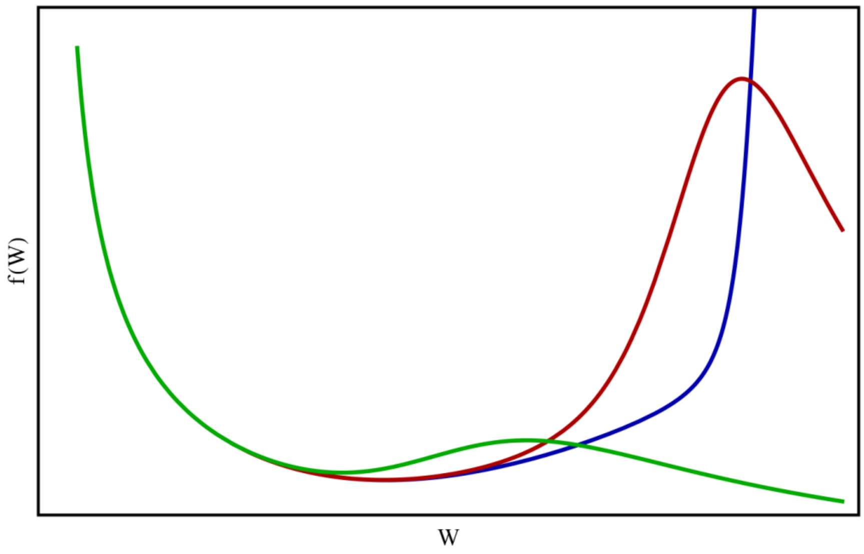

Supplementary figure 10: The function $\mathrm{f}(\widetilde{W})$ for $\nu = 0$, and $\widetilde{W} < \widetilde{W}_{\mathrm{crit}}$ at different values of $\alpha = 0.01(blue), 0.1(red)$,1 (green). The function is non-monotonic in $\widetilde{W}$. Its increase towards $\widetilde{W} = 1$ indicates softening to pitch variation. This abnormal softening decreases as α increases.

Comparing the theoretical values to the experimental ones is done by binning the measurements of twisted ribbons (pitch) only. We divided it into 50 bins, with overlap (moving average) for each bin we calculate the mean value and the standard deviation. Since $f(W)$ is known (once we have an estimate of *(t, W,*α, ν, k_0*)*, we divide the experimental values by the local theoretical value. Fitting is then done using a single parameter (Y). We also verify our results, by normalizing the energy at every given width into a dimensionless variable, $\Delta\widetilde{\mathrm{E}}$. If the distributions in all widths were Gaussian, this should result with a Gaussian distribution with a *std* of 1 for all widths. Plotting it in a semi-log (y axis) plot vs. $\Delta\widetilde{\mathrm{E}}$ should result with a straight line with slope one. This is shown in Fig. 4b in the main text.

Expected skewness: Our experimental data indicate positive skewness of the pitch distribution (Fig. 4b inset). We present experimental indications that similarly to the *std*, the skewness of the distribution is non-monotonic in W; first decreasing, as in compatible slender objects, but then increases as W approaches the twist-to-helical transition width. We calculate the skewness predicted for ribbons with the geometrical parameters (t, ν, k_0, α), used throughout the text and the young's modulus $Y = 9.5\ MPa$, determined from the *std* at room temperature (Fig. 11). The curve is consistent, both is trend and in magnitude with the measured data. However, meaningful quantitative comparison requires more experimental data.

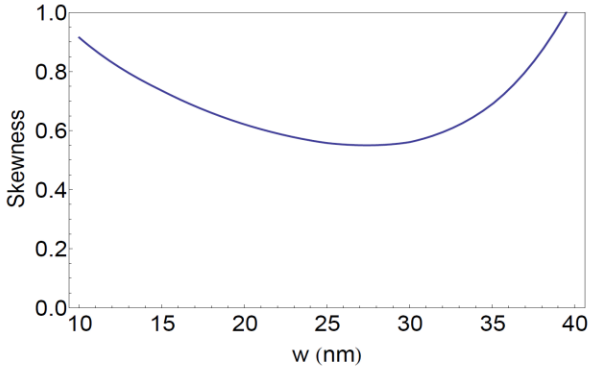

Supplementary figure. 11: The computed skewness of the pitch distribution vs. W, calculated using the parameters $k_0 = 0.03\ \frac{rad}{nm}$, $\alpha = 0.1, t = 3.4\ nm$, $\nu = 0.50$ and $Y = 9.5\ MPa$.

Compatible toy model: In contrast to our incompatible model (Eq. 3 in main text) we may consider a compatible model (here we include the exact numerical pre-factors that should appear also in Eq. 3 in the main text) whose energy is given by:

$$E = \frac{tW^5}{80}\left(\frac{1}{P^2} - \frac{1}{P_0^2}\right)^2 + \frac{t^3 W}{3}\left(\frac{1}{P} - \frac{1}{P_0}\right)^2$$

In such a model there is always a zero energy minimum $P = \frac{1}{k_0}$. As a ribbon gets wider its rigidity grows, the energy minimum becomes sharper and deeper. As a result, fluctuation become smaller and more Gaussian (the low temperature approximation gets better and the Skewness approaches zero), in clear contrast to the results of our experiment.

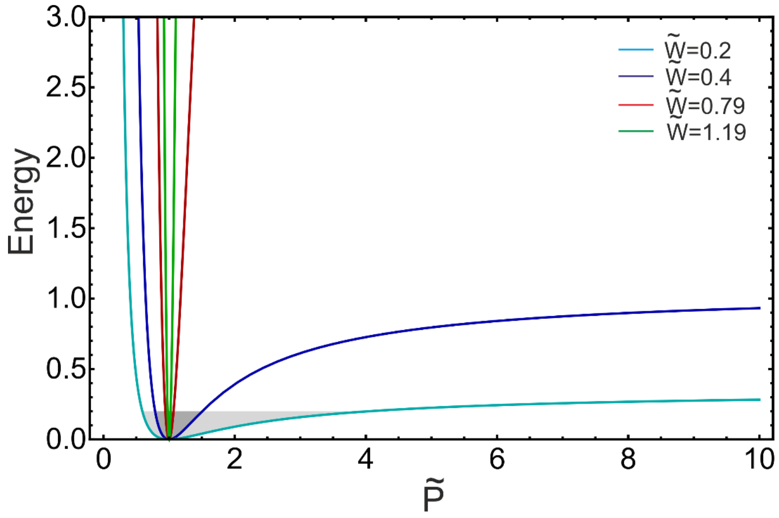

Supplementary figure 12: Dimensionless energy vs. pitch in the *compatible* toy model for $k_0 = 1$, and different values of $\widetilde{W}$ (indicated) as in Fig 4d (main text). Grey areas mark the region accessible by fluctuations at a fixed dimensionless thermal energy, $\widetilde{k_b T} = 0.2$. The variation of the energy curves with $\widetilde{W}$ suggests that as $\widetilde{W}$ grows the *std* and skewness of the pitch distribution are expected to decrease.

5) Modeling other types of assemblies: Here we demonstrate how to apply our approach to several different scenarios. Our main aim is to show how to integrate information on the molecular level, through the definition of the continuum reference geometry, and finally stating the solvable elastic equations. We do not solve these models, but comment on characteristics of the solutions. It is important to note that unlike the study in the main text, here we do not perform the analysis in the molecular level, but only assume hypothetical (though reasonable) cases.

Symmetric bi- layer of chiral amphiphiles: This example is relevant for a wide range of systems such as (*11, 12*). In such systems a **bi-layer** is formed due to hydrophobic interactions as well as non-covalent interactions, and the assembled molecules are chiral. Similarly to the C12-β12 system, there are **no lateral gradients**, so the reference metric is flat: $\bar{a} = \begin{pmatrix} 1 & 0 \\ 0 & 1 \end{pmatrix}$ and the chirality of the head groups induces a twist along the x direction. However, unlike C12-β12, the bi-layer's *faces are identical* (up to rotation) thus **we have** $\boldsymbol{\alpha = 0}$. And the expression for the reference curvature is:

$$\bar{b} = \begin{pmatrix} 0 & k_0 \\ k_0 & 0 \end{pmatrix}$$

The value of k_0 is obtained as in section 3: $k_0 = \frac{\theta_{eq}}{D}$, where $\theta_{eq} = \frac{\gamma D^2 \theta_0}{\gamma D^2 + \beta L^3}$. As in the main text, D, L, β, γ are the molecular distance, hydrophobic chains' length, hydrophobic interaction strength and head group interaction strength, respectively. The angle θ_0 is the close packing angle between two head groups. One can then proceed with solving the resultant elastic equations, as described in the main text. This case was solved in (*9*) and in (*10, 13*). In this case the transition between twist to helical is **a sharp transition** (rather than a smooth one).

It is important to note (as mentioned in the text) that θ_{eq} can be obtained much more accurately from a simulation of two-molecule interaction, a method that is not used in the present work. The benefit in the approximated expression above is that it provides the **functional dependence** of k_0 on the molecular parameters, thus can be used to guide the **design** of molecules for a desired suprastructure. For example: controlling L, the carbon chain length, can be used in order to **tune** $\boldsymbol{k_0}$ **and** $\boldsymbol{t}$, thus controlling tube diameter and helix pitch.

Asymmetric bi- layer of non-chiral amphiphiles: Consider a range of **asymmetric** membrane forming molecules (see illustrations in Fig. S13 a). As a specific example, one may consider an asymmetric bilayer, and neighboring molecules form **isotropic**, homogenous, bond structure. As before, the absence of lateral gradients implies a flat reference metric: $\bar{a} = \begin{pmatrix} 1 & 0 \\ 0 & 1 \end{pmatrix}$. Isotropy (and the lack of any chirality) also suggest that $\bar{b} = \begin{pmatrix} k_0 & 0 \\ 0 & k_0 \end{pmatrix}$, i.e., the reference curvature describes a surface of constant positive Gaussian curvature. If the typical distance (in the case of close packing, this is the typical size of a molecule) between molecules on one leaflet is R_1 and on the other it is R_2, then the typical curvature is given by $k_0 = \frac{1}{t} \frac{R_1 - R_2}{R_1 + R_2}$, where t is the thickness of the sheet. This kind of system was studied theoretically in [Grossman et. al. Phys. Rev. E, 022502, 2018] and was found to exhibit unique shape transition between a ring-shape and a set of *degenerate configurations* all are *helices*

with different pitch, radius and handedness (see Figure 13 b). Thus, in such systems one would **not expect a one to one relation between W and P.**

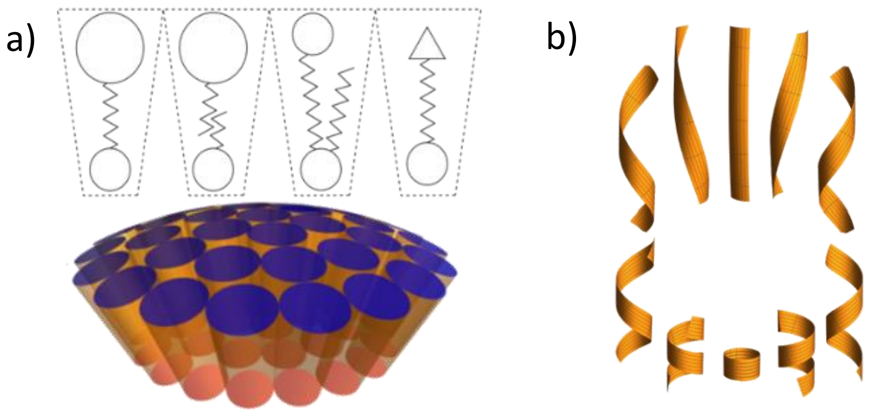

Supplementary figure 13: A) Different Molecular geometries that give rise to a positive reference curvature. b) Different possible ribbon configurations that have the same energy (degenerate)**.**

Environmental effects via change in strength of interactions: Different solvents and pH differences (together with temperature and other effects) are known to change the strength of molecular interactions (by modifications of hydrophobicity, electrostatic screening, etc.). Here we sketch how to derive a possible effect of these processes on the shape of a self-assembled chiral ribbons. We consider as an example the case of a symmetric bi-layer, as discussed above (example a). Consider an environmental change, which is translated into a change in the *typical distance* between molecules (Such information must come from chemical analysis or a simulation of the two-molecule interactions). For concreteness, let's consider a case when the typical distance between molecules increases. Such an effect will not change $\bar{a}$ – the sheet is still Euclidean - but will change $\bar{b}$ in two ways:

a. As we discussed in the main text, for a given twist angle θ_{eq}between neighboring molecules, we have $k_0 = \frac{\theta_{eq}}{D}$. Hence, larger D will give rise to **smaller** k_0.
b. Change in intermolecular distance will also change θ_{eq}. The fact the molecules are further away suggests that the interaction "spring constant" (γ) between neighboring head groups, thus $\theta_{eq} = \frac{\gamma D^2 \theta_0}{\gamma D^2 + \beta L^3}$, gets **smaller** as well.

We therefore predict **a decrease in k_0** , which will be manifested in an increase in the pitch and radius of helical configurations, the increase in tube diameter, and **can even lead to complete flattening of the ribbons** and the formation of wide sheets.

Ribbons with Lateral gradient: So far we discussed cases in which there are no lateral gradients. However, this is not always so. Some ribbons can be formed via lateral assembly of a single layer of amphiphiles. Consider the case of a single layer of amphiphiles in which the sizes of the groups that form the amphiphile are highly different, or where the in-plane bonds between molecules change throughout the ribbon (width, or length). In such cases the reference metric, $\bar{a}$ **, is no longer Euclidean**.

We illustrate such a scenario in the figure S14 below, for the case where the groups along the edge are larger than those in the interior. Here D, d, W are the edge and interior groups diameters and the ribbon width, respectively. The reference Gaussian curvature along the ribbon's center-line is: $\bar{K} \approx \frac{d-D}{(d+D)W^2}$. For the example illustrated below, this indicates $\bar{K} < 0$. This expression, together with $\bar{\boldsymbol{b}} = \mathbf{0}$ are the inputs to the energy functional in (*9*), which can be solved analytically. Related systems were studied in (*14*).

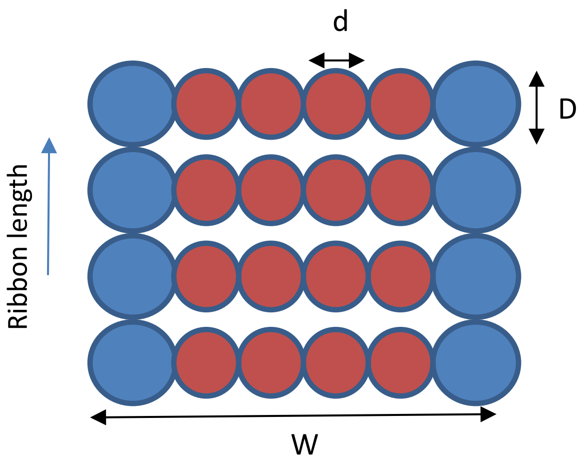

Supplementary figure 14: An illustration of the faces of a ribbon assembled along the (vertical direction) with lateral gradients. The relevant dimensions are marked.

Rods: Finally, many systems form rods - long filaments that bundle together – rather than ribbons. Such systems might be frustrated as well. The elastically of such rods is studied within incompatible elasticity theory in (*15*), where the emergence of twist and bend from the micro structure is presented. As before, chemical analysis or molecular simulations are needed in order to determine the relevant lengths and angles. These, must be fed into the relevant elastic energy functional in order to obtain solutions for the shape and (possibly) statistics.

Supplementary References

1. I. S. Radzishevsky *et al.*, Structure-activity relationships of antibacterial acyl-lysine oligomers. *Chem. Biol.* **15**, 354-362 (2008).
2. I. S. Radzishevsky *et al.*, Improved antimicrobial peptides based on acyl-lysine oligomers. *Nat. Biotechnol.* **25**, 657-659 (2007).
3. L. Ziserman, H.-Y. Lee, S. R. Raghavan, A. Mor, D. Danino, Unraveling the Mechanism of Nanotube Formation by Chiral Self-Assembly of Amphiphiles. *Journal of the American Chemical Society* **133**, 2511-2517 (2011).
4. A. Gavezzotti, *Acc. Chem. Res.* **27**, 309-314 (1994).
5. A. Gavezzotti, G. Filippini, *J. Phys. Chem.* **98**, 4831-4837 (1994).

6. N. Nandi, B. Bagchi, Molecular origin of the intrinsic bending force for helical morphology observed in chiral amphiphilic assemblies: Concentration and size dependence. *Journal of the American Chemical Society* **118**, 11208-11216 (1996).
7. C. F. Macrae *et al.*, Mercury CSD 2.0 - new features for the visualization and investigation of crystal structures. *J. Appl. Crystallogr.* **41**, 466-470 (2008).
8. E. Efrati, E. Sharon, R. Kupferman, Elastic theory of unconstrained non-Euclidean plates. *Journal of the Mechanics and Physics of Solids* **57**, 762-775 (2009).
9. D. Grossman, E. Sharon, H. Diamant, Elasticity and Fluctuations of Frustrated Nanoribbons. *Physical Review Letters* **116**, 5 (2016).
10. S. Armon, E. Efrati, R. Kupferman, E. Sharon, Geometry and Mechanics in the Opening of Chiral Seed Pod. *Science* **333**, 1726-1730 (2011).
11. R. Oda, I. Huc, M. Schmutz, S. J. Candau, F. C. MacKintosh, Tuning bilayer twist using chiral counterions. *Nature* **399**, 566-569 (1999).
12. E. T. Pashuck, S. I. Stupp, Direct Observation of Morphological Tranformation from Twisted Ribbons into Helical Ribbons. *Journal of the American Chemical Society* **132**, 8819-8821 (2010).
13. S. Armon, H. Aharoni, M. Moshe, E. Sharon, Shape selection in chiral ribbons: from seed pods to supramolecular assemblies. *Soft Matter* **10**, 2733-2740 (2014).
14. E. Efrati, E. Sharon, R. Kupferman, Hyperbolic non-Euclidean elastic strips and almost minimal surfaces. *Physical review. E, Statistical, nonlinear, and soft matter physics* **83**, 046602 (2011).
15. H. Aharoni, Y. Abraham, R. Elbaum, E. Sharon, R. Kupferman, Emergence of Spontaneous Twist and Curvature in Non-Euclidean Rods: Application to Erodium Plant Cells. *Physical Review Letters* **108**, (2012).
